# The Treatment of Sciatica

**Published:** 1904-10-22

**Authors:** 


### THE TREATMENT OF SCIATICA.

Some recent suggestions on the treatment of sciatica may, perhaps, be usefully collected and noted. Dr. J. Farquhar[1] has found both in sciatica and lumbago "when metabolic in origin, or symptomatic of the rheumatic habit" that pilocarpine proves a sheet-anchor. He uses one-third of a grain of the nitrate injected subcutaneously every night or every second night, and selects painful points as the sites for puncture. He also orders a minim of croton oil and a saline purge once a week. In the "most hopeless" case he has experienced this method of treatment proved successful in three weeks. Cardiac disease is suggested as a bar to the use of pilocarpine. Mr. Arthur H. Bostock,[2] after a thorough trial, advises that in the acute stage the patient should be kept in the recumbent posture and poultices should be applied. Phenalgin he finds to be the best internal remedy. He orders this in a 10-grain dose every three hours for 24 hours, and then gradually reduces the amount. This drug acts better in purely neuralgic cases than in those associated with the rheumatic diathesis. Dr. T. Churton[3] emphasises the value of acupuncture. He recommends that after thorough sterilisation of the skin of the buttock a long acupuncture needle should be passed down to the bone in the position of the nerve an inch or less above the level of the great trochanter, and a second needle be placed two or three inches lower, the needles being left in position for 20 minutes. Dr. Hamilton Hall[4] finds evidences of gout, in the shape of a hard pulse, in some cases of sciatica, and on this view has used colchicum with free purgation He is able to relate good results from this treatment. Dr. W. F. Somerville[5] has had considerable experience of high-frequency currents in the treatment of sciatica and lumbago. He reports a number of cases in which very prompt relief was obtained, and, tracing his patients for some months, and in a few instances for years, he finds that the cure is usually permanent. Dr. Charles B. Lawrence[6] relates the case of a man of 52 years who had suffered severely from sciatica for seven weeks, and who had been compelled to take morphine freely to relieve the pain. Various forms of treatment had been tried, but all had proved ineffectual. It was then determined to try the administration of nitro-glycerine. One minim of an alcoholic solution was ordered to be taken three times a day, and the dose gradually increased to five minims. Improvement was almost immediate, and in ten days the patient was able to walk to his work. A complete recovery ensued.

[1] Brit. Med Jour., April 16, 1904. [2] Lancet, April 16, 1904. [3] Brit. Med. Jour., Sept. 10, 1904. [4] Ibid. [5] Jour. of the British Electro-Therapeutic Society. [6] Clinical Jour., Vol. II., p. 304.

